# Modelling the Dynamics of Feral Alfalfa Populations and Its Management Implications

**DOI:** 10.1371/journal.pone.0039440

**Published:** 2012-06-29

**Authors:** Muthukumar V. Bagavathiannan, Graham S. Begg, Robert H. Gulden, Rene C. Van Acker

**Affiliations:** 1 Department of Plant Science, University of Manitoba, Winnipeg, Manitoba Canada; 2 The James Hutton Institute, Invergowrie, Dundee United Kingdom; 3 Department of Plant Science, University of Manitoba, Winnipeg, Manitoba Canada; 4 Department of Plant Agriculture, University of Guelph, Guelph, Ontario Canada; East Carolina University, United States of America

## Abstract

**Background:**

Feral populations of cultivated crops can pose challenges to novel trait confinement within agricultural landscapes. Simulation models can be helpful in investigating the underlying dynamics of feral populations and determining suitable management options.

**Methodology/Principal Findings:**

We developed a stage-structured matrix population model for roadside feral alfalfa populations occurring in southern Manitoba, Canada. The model accounted for the existence of density-dependence and recruitment subsidy in feral populations. We used the model to investigate the long-term dynamics of feral alfalfa populations, and to evaluate the effectiveness of simulated management strategies such as herbicide application and mowing in controlling feral alfalfa. Results suggest that alfalfa populations occurring in roadside habitats can be persistent and less likely to go extinct under current roadverge management scenarios. Management attempts focused on controlling adult plants alone can be counterproductive due to the presence of density-dependent effects. Targeted herbicide application, which can achieve complete control of seedlings, rosettes and established plants, will be an effective strategy, but the seedbank population may contribute to new recruits. In regions where roadside mowing is regularly practiced, devising a timely mowing strategy (early- to mid-August for southern Manitoba), one that can totally prevent seed production, will be a feasible option for managing feral alfalfa populations.

**Conclusions/Significance:**

Feral alfalfa populations can be persistent in roadside habitats. Timely mowing or regular targeted herbicide application will be effective in managing feral alfalfa populations and limit feral-population-mediated gene flow in alfalfa. However, in the context of novel trait confinement, the extent to which feral alfalfa populations need to be managed will be dictated by the tolerance levels established by specific production systems for specific traits. The modelling framework outlined in this paper could be applied to other perennial herbaceous plants with similar life-history characteristics.

## Introduction

Alfalfa is an important forage crop in the Northern Great Plains of North America, and has the potential to establish in competitive environments without managed cultivation [1], [2]. Alfalfa is shown to withstand grass competition and some alfalfa populations are known to have been persistent for several decades in pasture lands [3]. Feral alfalfa populations are commonplace in roadside environments in alfalfa growing regions. The existence of roadside alfalfa populations has been documented in Europe [4], and in North America [5], [6]. Alfalfa has found its way to roadside habitats possibly through seed escape during transport and farming activities, intentional planting during road construction for stabilising the roadsides and/or in some cases, planting by farmers for haying. Whatever the source, alfalfa is capable of persisting in the roadsides often as self-sustaining populations [2].

The existence of feral alfalfa populations in roadside habitats has implications for novel trait confinement because feral populations constitute an important component of the metapopulation of cultivated crops in the agricultural landscapes [7]. Alfalfa is a highly outcrossing species and pollen-mediated gene flow between cultivated and feral populations can be facilitated by pollinating insects. Genetically-engineered (GE) alfalfa has been approved for environmental release in North America and efficient trait confinement protocols can greatly benefit stakeholders for achieving specific tolerance levels, if any, warranted by specific production systems. Efficient protocols for novel trait confinement in alfalfa will consider the role of feral populations in novel trait movement for a given production situation [8]. Despite the significance of feral alfalfa populations in trait movement, the long-term dynamics of these populations have not been described.

Understanding the long-term dynamics of plant populations is fundamental to devising strategies for their management [9]. In particular, knowledge on vital growth rates and the effects of interventions on the population behaviour is instrumental for making appropriate management decisions [10]. Projecting the population dynamics beyond the few years of observation has been the primary focus for most plant demographers, and mathematical models are useful in simulating the long-term dynamics of plant populations based on available empirical data. Simulation models allow us to describe complex population dynamics using simple demographic descriptors and hypothetically test the possible consequences of changes in vital growth rates, past or future changes in management interventions or changes in the environment, on the long-term population dynamics [11]. In particular, matrix based models have been commonly used in theoretical and applied studies in plant population ecology [11]–[13], in part because of their versatility and broad applicability [14], [15].

In agroecology, population dynamic models have often been used to understand the demographic processes of weed populations under diverse production systems [16], [17]. The use of population dynamic models in agroecological research has steadily increased in the past decade, especially in connection with the large-scale introduction of GE crops. In particular, population dynamic models have been widely used in understanding the dynamics of feral/volunteer crop populations to aid novel trait confinement protocols [18]–[22], or to simulate the evolution of herbicide resistance in weed populations for devising suitable resistance management strategies [23], [24].

We developed a matrix based population dynamic model encompassing density dependence and recruitment subsidy for roadside feral alfalfa populations occurring in southern Manitoba, Canada, with an aim to identify strategies for mitigating feral population mediated novel trait movement in this species. Based on a previous assessment of the demography of these populations [2], the present study investigates their long-term dynamics, taking into account the existing road-verge management practices such as mowing and herbicide application. The following specific questions were addressed: a) what are the long-term dynamics of feral alfalfa populations? b) what impact does mowing or herbicide application have on the persistence of feral alfalfa?, and c) what are the management implications with regard to minimising novel trait movement?

## Methods

### Study Species

**Figure 1:**
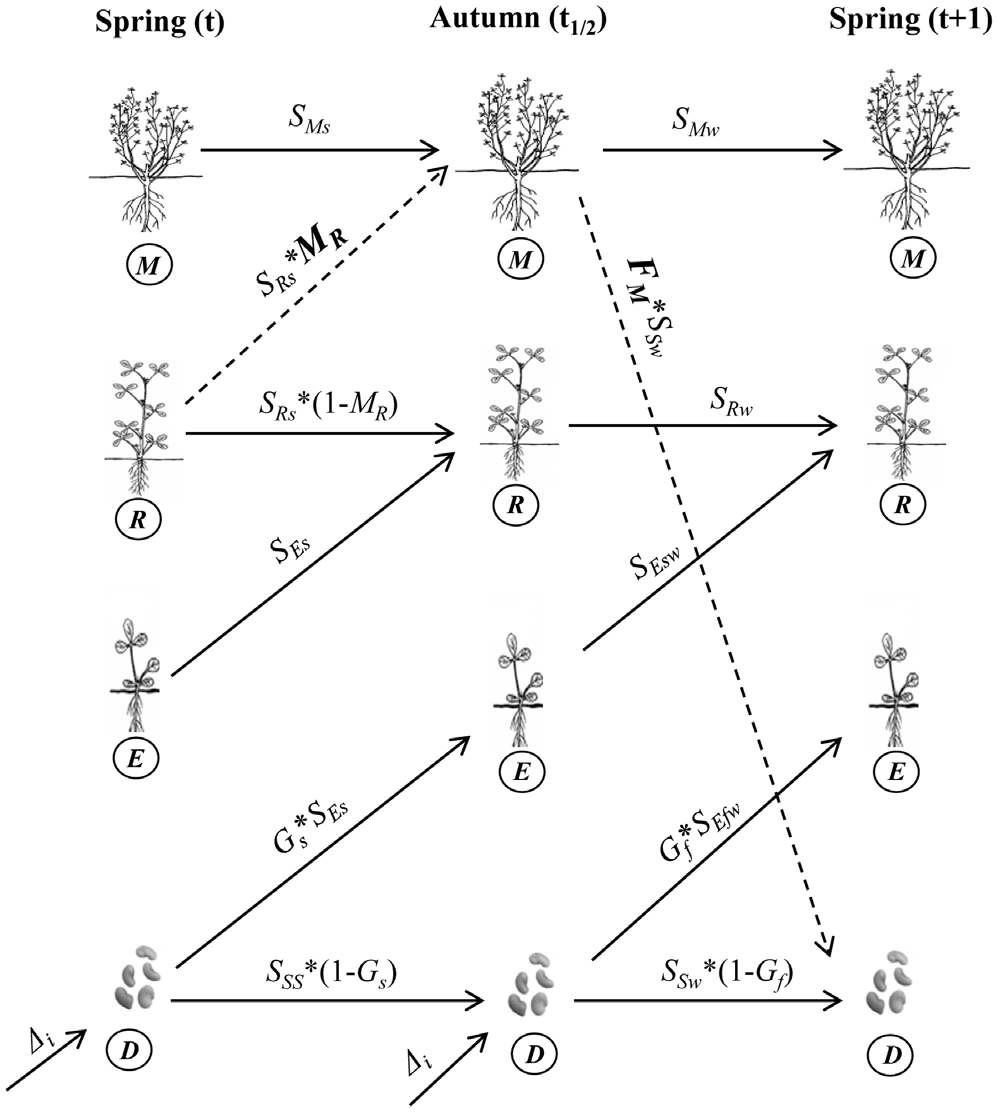
Life cycle diagram of feral alfalfa used in defining the transitions. It comprises of four distinct stages including dormant seeds in the seedbank (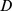), emerged seedlings (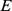), rosettes (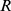) and adult plants (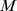), and two time periods namely spring (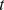) and autumn (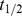). The dashed arrows illustrate reproductive transitions. The function associated with each arrow corresponds to the transition coefficient from 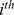 stage to the 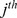 stage and the density dependant parameters in the functions are denoted by bold letters. The diagram includes an immigration component 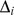 which represents the seed input into the population from external sources (i.e. recruitment subsidy). Seeds in the seedbank either germinate [spring (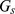), autumn (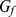)] or remain in the seedbank via survival [summer (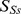), winter (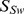)]. Seedlings recruited in spring survive during summer (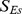) and winter (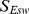) to form rosettes the following spring. The seedlings recruited in autumn survive the winter (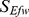), survive the following summer (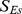) and become rosettes. Rosettes survive [summer (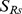), winter (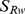)] and transform to adult plants (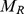) upon flowering, which occurs during mid to late summer. Adult plants produce seeds during autumn (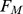) and the seeds that survives winter (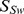) contributes to the seedbank levels in the following spring. Adult plants persist in the environment through summer (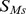) and winter survival (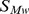).

Alfalfa has four distinct stages in its lifecycle: seeds, seedlings, rosettes, and mature plants (i.e. adult plants). The demographic processes within a single population of alfalfa are illustrated in Figure 1. In roadside habitats, alfalfa seedlings usually emerge in early spring and early autumn, although spring recruitment is the predominant one [25]. Seedlings recruited in spring develop into well established seedlings in the summer when they can be easily identified by the presence of cotyledons. Spring recruited seedlings over-winter and become rosettes (the vegetative phase of the lifecycle) during the following spring. Seedlings recruited in autumn survive the winter, seedlings re-establish in spring and become rosettes in the following autumn. However, winter mortality is typically high in autumn-emerged seedlings [25]. This is succeeded by the adult stage, which plants enter upon initiation of flowering. Based on our observation in roadside feral populations, flowering rarely occurs in the same year as seedling recruitment and plants typically take more than one year to reach adult stage. Adult plants flower in mid- to late-summer, produce seeds, and the shoots subsequently die-off. The rosettes and adult plants can regrow in the spring from over-wintering crowns.

Alfalfa is an indeterminate plant, exhibiting a prolonged flowering and pollination period. Alfalfa possesses high levels of self-incompatibility, making it predominantly outcrossing. Pollination is usually facilitated by insects such as bees that are capable of tripping the flowers. Alfalfa plants can grow robust and produce thousands of seeds per plant. Seeds fall within a meter of the mother plant, and the establishment of new seedlings is often affected by the presence of auto-allelopathy [26]. Alfalfa seeds possess hard seed coat, and reports indicate that they can remain viable in the soil for several decades. A detailed review of the ecology and biology of this species was prepared by Bagavathiannan and Van Acker [1].

### Study Area and Site Selection

The study was conducted between 2006 and 2009 on roadside alfalfa populations identified in three rural municipalities (RMs) in southern Manitoba, Canada. These RMs include Hanover (49°28′ N, 96°50′ W; area = 718 km^2^), MacDonald (49°40′ N, 97°30′ W; area = 1,059 km^2^), and Springfield (49°55′ N, 96°45′ W; area = 1,106 km^2^). Southern Manitoba is characterised by cold winters and warm summers, with an average seasonal temperature ranging from −13°C (winter) to 26°C (summer). Among the RMs, alfalfa is widely grown in Hanover (11% of total cultivated area), which is followed by Springfield (4%) and MacDonald (<1%) [27]. In each RM, four roadside alfalfa populations were randomly selected, and in each population, 30 alfalfa plants were chosen for collecting the demographic data. Roadside management, particularly mowing and herbicide application, is common in this region. The roadsides are usually mown twice each year at a mower height of about 20 cm, with the first mowing occurring between early-June and early-July and the second mowing (to manage regrown plants) between late-August and late-September. In most cases, only the area adjacent to the road shoulder (about 3.5 m wide) is mown (i.e. *mown strips*), while the alfalfa plants occurring further from the road remain largely unaffected by mowing (i.e. *non-mown strips*). The pattern of mowing varies greatly within and among the municipalities. In addition, the roadsides are sometimes sprayed with the herbicide 2,4-D (with or without dicamba) as part of the noxious weed management program by the RMs. The study sites were ideal for understanding the demography of feral alfalfa populations and evaluating the impacts of roadside management regimes on their dynamics. No specific permits were required for the described field studies, locations/activities, but the respective RM roadverge managers and weed supervisors were informed of our study locations in order to protect the sites from any unintended disturbance. The study locations were not privately-owned or protected in any way.

### Model Description

To analyse the population dynamics of feral alfalfa, a stage-structured matrix projection model was constructed, representing the stage-specific demographics described above. The matrix projection model was constructed as per Caswell [12], and the general framework of the model is described as follows,
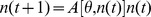
where the state vector 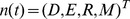, the density of individuals (m^−2^) in each of the four developmental stages (seeds, seedlings, rosettes, and mature plants, respectively; see Figure 1) at time 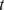, 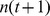 is the state vector at the next time step (i.e. 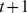), and A is the population projection matrix which depends on 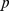 parameters represented by the 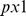 vector 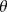. The initial colonisation of road verges by alfalfa demonstrates the capacity for immigration by seed dispersal (denoted by 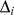 in Figure 1), facilitated by farming activities such as seeding, harvesting and transport operations. The effect of the recruitment subsidy resulting from seed immigration is captured in the model by the addition of the vector 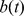 in which all elements are set to zero with the exception of the first, which contains the density of seeds entering the population from external sources,



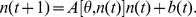



For ease of construction, the transition matrix 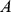 was represented by two seasonal transition matrices, i.e.
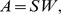
where 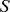 and 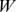 are the transition matrices corresponding to over-summer (spring to autumn) and over-winter (autumn to spring) transitions respectively. In the context of this paper, spring and autumn refers to the time point immediately before spring and autumn seedling recruitment.

From Figure 1, we can derive the elements of the matrices 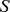 and 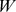 as follows:
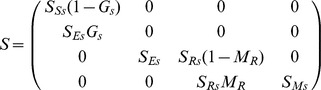


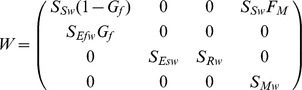
Here, 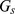 and 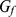 combine germination with emergence for spring and autumn, respectively. Summer and winter survival of seeds 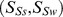, rosette 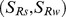 and adult plants 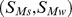 are denoted by respective symbols. 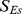 is the proportion of seedlings that survived summer, while 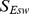 and 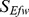 represent winter survival of the seedlings recruited in spring and autumn, respectively. 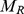 is the proportion of rosettes that transition into adult plants and 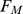 is the fecundity of adult plants. It is assumed that all winter-surviving seedlings transform to the rosette stage in the following spring.

It is likely that density-dependent processes operate in roadside alfalfa populations because of resource limitation, short range seed dispersal, and the presence of auto-allelopathy. Data from a previous field study of alfalfa demographics [2] and other published sources were examined for evidence of density-dependence between individual model parameters and total and stage specific plant densities. The possibility of density-dependence was considered for all parameters, but was only identified in the response of over winter survival of spring emerged seedlings, 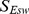, and plant fecundity, 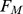, to adult plant density, 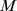.

The density-dependent response of 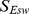 was represented by a logistic function of the form,
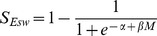
which ensures that the values for 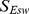 are bounded between 0 and 1 and where 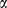 and 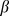 are parameters that determine the shape of the relationship.

Plant fecundity, 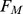, showed an exponential decline in response to increasing densities of mature plants, i.e.,
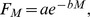
where, 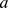 and 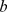 are parameters that determine the shape of the relationship, 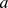 being the maximum fecundity in the absence of other plants and 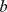 is the strength of the density-dependence.

### Parameter Estimation

Field experiments conducted in the southern Manitoba region (see *Study area and site selection*) provided the majority of the demographic data required for parameter estimation. The parameters of the density-dependent function of 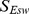 were estimated by logistic regression procedures using the data obtained from literature [3]. The density-dependent fecundity, 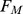, was estimated using the log transformed fecundity data [28]. All parameter estimates are provided in Table 1.

**Table 1:** The parameter values used in the population dynamic model for roadside alfalfa.

Parameter	Symbol	Non-mown	Mown	Reference
*Seed summer survival£	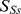	0.35	0.35	–
Seed winter survival£	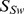	0.81	0.81	[2]
Seedling summer survival	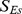	0.85	0.79	[2]
Seedling winter survival (spring germinated)†	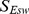	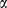 = 0.20 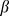 = 0.65	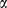 = 0.20 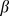 = 0.65	[28]
Seedling winter survival (autumn germinated)	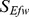	0.12	0.12	[2]
Rosette summer survival	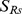	0.99	0.99	[2]
Rosette winter survival	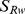	0.72	0.51	[2]
Adult plants summer survival	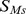	0.96	0.95	[2]
Adult plants winter survival	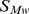	0.98	0.95	[2]
Spring seed germination	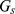	0.05	0.05	[2]
Autumn seed germination	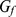	0.04	0.01	[2]
Transition of rosettes to adult plants	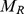	0.41	0.63	[2]
Fecundity of adult plants†	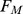	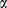 = 4000 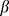 = 0.18	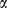 = 600 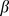 = 0.18	[3]
*Recruitment subsidy	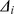	0 (or) 1500	0 (or) 1500	–

† denotes density-dependant parameter.

£ values after accounting for seed herbivory, decay and other likely forms of seed loss from the system.

*parameter values based on expert assumptions.

### Management Scenarios

Parameter values were estimated separately to independently simulate the dynamics of the mown (plants regrow after mowing) and non-mown portions of the alfalfa populations. For the mown strip, two different scenarios were assumed. The first being the existing mowing regime, which allowed some seed production in plants regrown after first mowing, as observed in our field experiment [2]. The other scenario assumed a timely mowing scenario (a single mowing during early- to mid-August each year), wherein seed production is strictly prevented. The simulations also included a scenario with a single application (no subsequent applications in the following years) of a highly efficacious herbicide or herbicide mix. The herbicide application regime is not rigorous in roadside habitats, and it is less likely that the populations receive applications every year. If a full coverage is achieved, then all the demographic stages except dormant seedbank are eliminated from the starting population. However, it is possible that some individuals are left uncontrolled due to a lack of herbicide coverage and/or environmental conditions, among others. Therefore, we considered two situations: a) a complete elimination of seedlings, rosettes and adult plants, and b) 10% survival after herbicide applications (90% efficacy). Additionally, we tested the effect of herbicide application at different spray intervals to establish the minimum spray interval required to completely eliminate a feral population.

### Simulations

All simulations were initiated with a seedbank density of 100 seeds m^−2^ representing the colonisation of the roadside verge, and initial simulations showed results to be insensitive to the starting densities, as expected [12]. In simulations with recruitment subsidy, a constant annual immigration of 1,500 seeds m^−2^ was assumed to represent a worst-case scenario. In all cases, simulations were run for 100 years which initial simulations showed was sufficient for the behaviour of the model to have reached equilibrium for a wide range of parameter values.

### Model Analysis

The model analysis focused on the long-term dynamics under the different management scenarios, with or without subsidised recruitment. The sensitivity of this behaviour to parameter estimates was assessed by the construction of bifurcation diagrams which show the long-term behaviour of the model, in terms of the total spring population density from year 100 to 200 against a wide range of values for each parameter while assuming the other parameters were fixed at their base-line values. Where the parameter estimates resulted in equilibrium densities, the proportional sensitivity of the equilibrium to small changes in parameter values was assessed by elasticity analysis. Elasticity was calculated as described by Caswell [29] in which the elasticity in the equilibrium density of the *i*th stage in response to changes in the *j*th parameter, i.e.
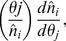
is given by



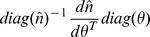



The model simulation and analysis were carried out in the ‘R’ programming environment version 2.12.1×64 [30].

## Results

### Feral Population Dynamics (No Recruitment Subsidy)

Model analysis revealed that roadside alfalfa populations typically reach equilibrium in the long-run. The absence of recruitment subsidy leads to equilibrium population densities for the mown (existing pattern) and non-mown strips of.
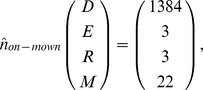


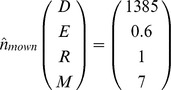



**Figure 2:**
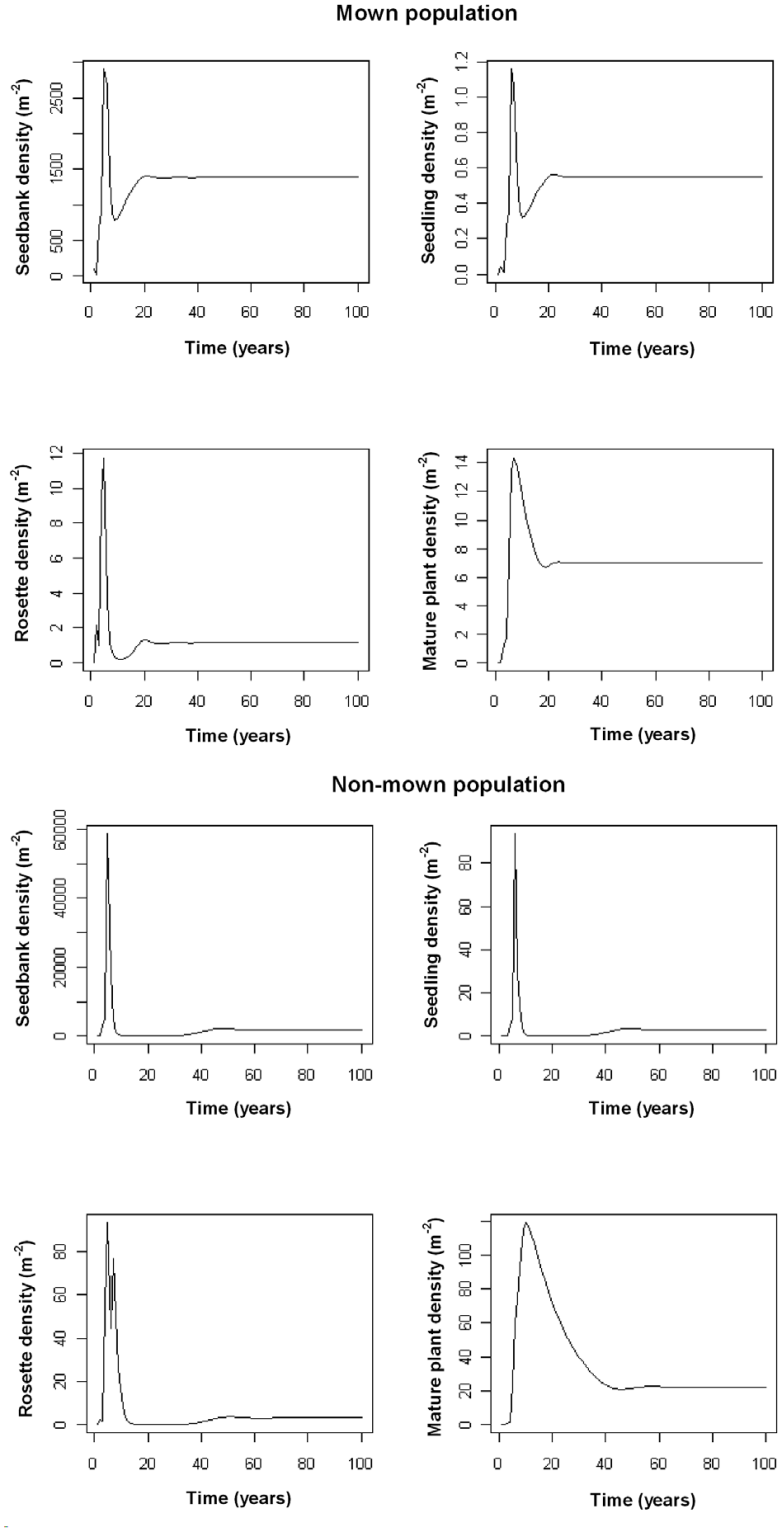
Long-term dynamics of different demographic stages of feral alfalfa populations under existing mowing and non-mown scenarios with non-subsidised, local recruitment. X axis indicates the duration of simulation (i.e. 100 years) and Y axis represents the abundance of individual demographic stages [i.e. seedbank, seedlings, rosettes, and adult plants].

In both scenarios, the populations are dominated by the seedbank but the results also show that the equilibrium densities are considerably lower for the mown strips, compared with the non-mown strips, the effect being most significant for the above ground life stages (Figure 2).

Mowing reduced the time taken for the adult plant populations to reach equilibrium, but increased the time taken for the other demographic stages. The time to equilibrium for the adult plants was about 25, and 45 years, respectively for the mown and non-mown scenario (Figure 2). The other demographic stages reached equilibrium within about 10 to 15 years in the non-mown strips, whereas it required about 25 to 30 years for the mown strips. Simulating a one-time herbicide application (either assuming complete seedling, rosette and adult plant mortality or allowing 10% of them to survive) did not affect equilibrium. However, a total control scenario with one-time herbicide application substantially delayed the time taken to reach equilibrium for the adult plants, with about 30 (mowing) or 55 (non-mowing) years to re-establish and reach equilibrium densities after the herbicide treatment. For the rest of the stages, the effects were similar to those of non-herbicide-treated scenario. In the absence of recruitment subsidy, a regular herbicide application regime at the shortest possible regeneration time (2 years) until the exhaustion of the seedbank (about 7 years) resulted in the complete elimination of the population. If any seed return is allowed, the population may re-establish and gradually move towards equilibrium densities. The timely-mowing scenario, which prevented seed production and seedbank renewal, eventually drove the populations to extinction. In this scenario, it took about seven years to nearly exhaust the seedbank and about 40 years to completely eliminate the population (all demographic stages).

**Figure 3:**
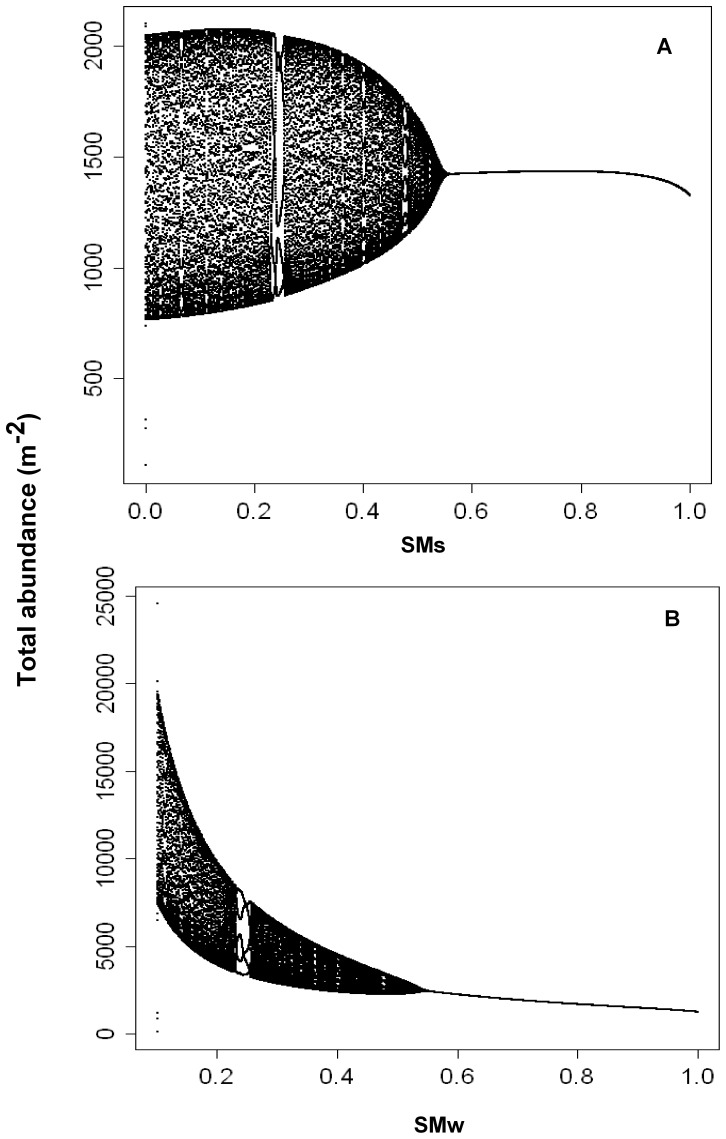
Bifurcation plots showing complex model behaviour: (A) summer survival (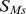), and (B) winter survival (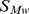) of adult plants under existing mowing pattern and non-subsidised, local recruitment. X axis represents the whole parameter range from 0 to 1 and Y axis indicates the total abundance (m^−2^) of all the different demographic stages [i.e. seeds, seedlings, rosettes, and adult plants].

A detailed bifurcation analysis indicated that for all scenarios the model reached a stable equilibrium across most of the parameter space. Exceptions to this were the summer (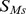) and winter survival (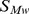) of adult plants for the mown strips, wherein a complex model behaviour was introduced when survival was below approximately 50% (Figure 3). Further, the bifurcation plots were similar for the herbicide treated scenario.

**Figure 4:**
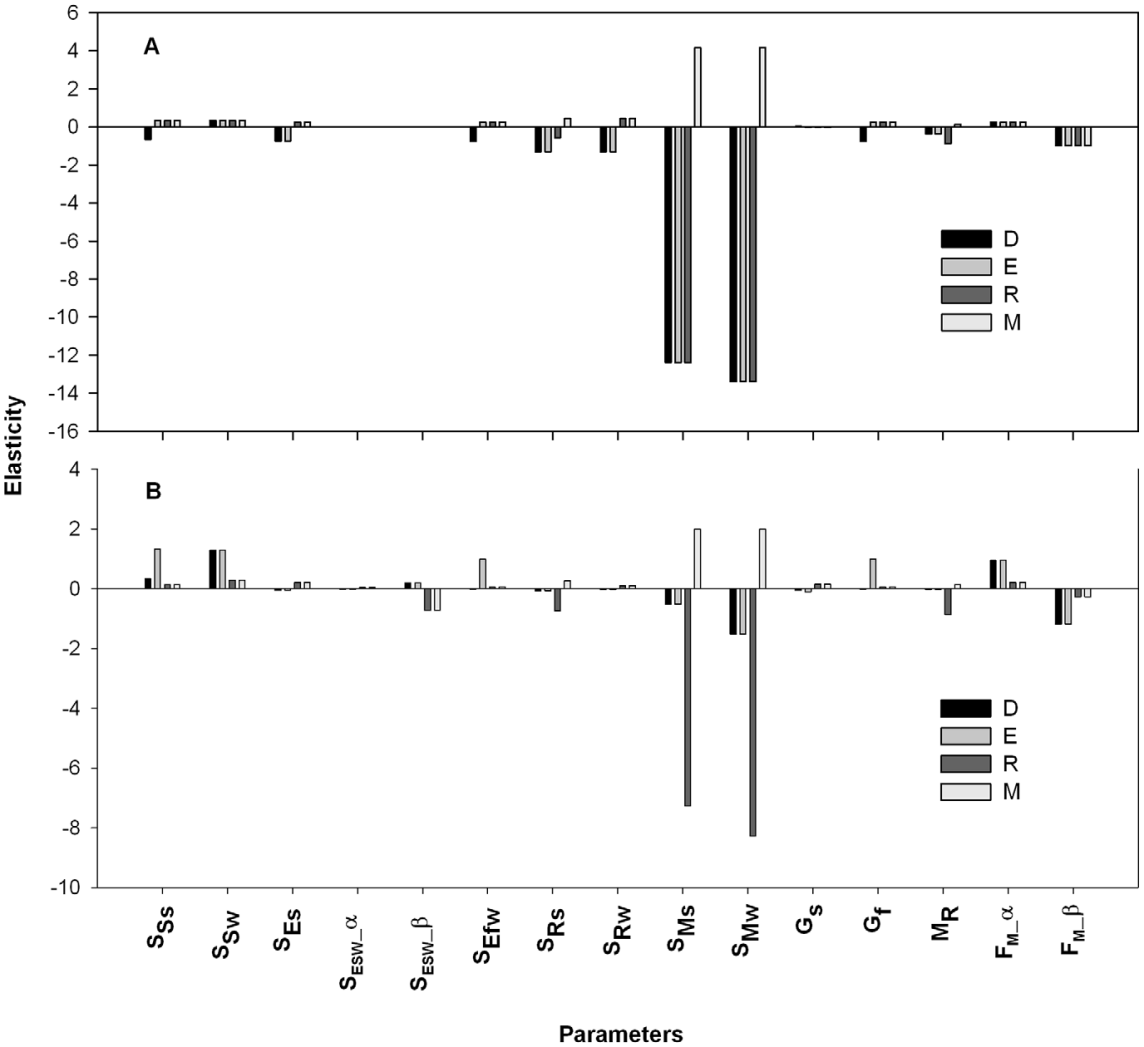
Elasticity of the density of each stage [i.e. seedbank (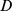), seedlings (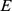), rosettes (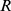) and adult plants (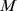)] with respect to each parameter at equilibrium. Elasticity analyses were performed for the scenarios, (A) non-mown and (B) mown (existing pattern), under non-subsidised, local recruitment. The parameters, including seed summer survival 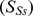, seed winter survival 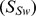, seedling summer survival 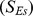, winter survival of spring recruited seedlings 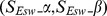, winter survival of autumn recruited seedlings 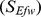, rosette summer survival 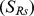, rosette winter survival 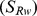, adult plant summer survival 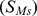, adult plant winter survival 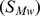, spring seed germination 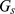, autumn seed germination 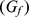, transition of rosettes to adult plants 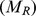, and fecundity of adult plants 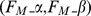 are denoted by respective symbols.

The elasticity analysis for the mown (existing pattern), and non-mown strips are presented in Figure 4. The elasticities for the summer (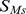) and winter (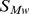) survival of adult plants were much larger than those for other parameters. The adult plant survival had a positive relationship with adult plant density (i.e. *M*), and an opposite relationship with the rest of the demographic stages (i.e. *D*, *E*, and *R*). This trend was similar for the mown, non-mown, and the herbicide scenarios. However, the elasticities for *D* and *E* were much reduced in the mown strips, compared with the non-mown strips (Figure 4).

### Feral Population Dynamics (Subsidised Recruitment)

A constant immigration of 1,500 seeds m^−2^ each year had a slight influence on the equilibrium densities (m^−2^) for the non-mown and the mown (existing pattern) scenarios, as follows:
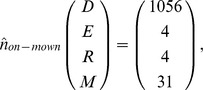


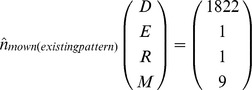



Overall, there was an increase in the densities of different stages in subsidised recruitment compared with no-subsidy, except for the seedbank density under the non-mown scenario. In the timely-mowing scenario, subsidised recruitment typically offset the failure in seed production by the local population, leading to the following equilibrium densities:
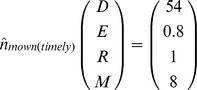



Compared with the non-subsidised scenario, there was a considerable increase in time to equilibrium for the adult plants, which were about 25, 30, and 60 years, respectively for the mown (timely), mown (existing pattern), and the non-mown scenarios. However, the time to equilibrium for the rest of the stages was not substantially influenced by recruitment subsidy. For the herbicide scenario, subsidised recruitment offset the prevention of seed production by herbicide application, eventually leading the population towards equilibrium, and the equilibrium densities were similar to that of non-treated strips. However, there was a substantial delay in time to equilibrium for the adult plants in the non-mown strips (about 65 years), particularly if all seedling, rosette and adult plants were eliminated by herbicide application.

The elasticity analysis revealed a trend similar to that of the non-subsidised setting, wherein 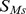 and 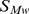 had larger elasticities than other parameters for all the management scenarios investigated. In addition, bifurcation analysis exhibited a complex behaviour for 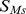 and 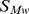 for the mown strips, while such a pattern was not observed for the non-mown scenario. Further, the bifurcation pattern in the herbicide scenario was similar to that of the mown strips.

## Discussion

The model suggests that roadside alfalfa populations can be persistent for the scenarios tested, and the likelihood of these populations becoming extinct is minimal under current road verge management regimes. Alfalfa has been shown to have a tremendous ability to establish and persist under competitive environments including roadsides [25]. Alfalfa cultivars are typically selected for persistence under grass mixtures, and the traits that favour their adaptation as a cultivated crop also favour their persistence in roadsides. In particular, the traits including, but not limited to, the ability to fix atmospheric nitrogen, presence of deep tap roots, drought and winter tolerance, perennial nature, high genetic diversity, and fast regrowth potential contribute to the persistence of alfalfa in competitive environments [1]. Moreover, hard seed coats of alfalfa seeds [31] lead to a persistent seedbank that can act as a reserve for re-establishing the population following adverse conditions [32] or catastrophic events, thus preventing extinction.

Feral alfalfa populations experience density-dependent regulation of population growth, allowing populations to reach equilibrium densities. Density-dependence is an important determinant of population growth, and therefore, is a key consideration to population dynamic models [33]–[35]. Although resource competition is the prime cause for density-dependence, the direction and magnitude may differ among different life history stages or environments [33]. Our field observations from roadside feral alfalfa populations suggest that greater density-dependent effects may be observed on the establishment of juveniles due to the existence of auto-allelopathy and limited dispersal abilities. In other species, density-dependent effects have been observed on seed germination [36], seedling establishment [37], and plant survival [38].

Recruitment subsidy facilitates the survival of feral alfalfa populations by compensating for any failure in seed production and local recruitment. Further, the effects of seed herbivory or other seed loss in native populations can be buffered by the inflow of propagules from external sources [39]. For feral *Brassica napus* populations in the UK, Crawley and Brown [40] showed that recruitment subsidy was an important process facilitating persistence. For populations of *Sorghum intrans* found in the wet-dry tropics, subsidised recruitment through the immigration of seeds from productive patches was an important mechanism for persistence [41]. The impact of recruitment subsidy on growth rates of feral alfalfa populations may vary among populations and environments. Nevertheless, emphasis on preventing seed immigration into roadside populations can be a useful strategy for preventing long-term persistence of these populations, especially if localised recruitment is limited. Total prevention of seed immigration can be a challenge. It has been shown that roadside traffic and agricultural vehicles can contribute to long-distance seed movement [42]; it is however a random event and the levels of seed immigration can vary.

Seed immigration generally increased the equilibrium densities, but was not an absolute requirement for sustaining the populations. As such, the results suggest that roadside alfalfa populations can be self-sustaining. An exception to this was the timely-mown scenario, in which the survival of the population was dependent on subsidised recruitment because the local seed production was completely arrested. Because seed immigration is a random and possibly infrequent event, it is less likely that plants in timely-mown strips can sustain their populations under such circumstances. Overall, it appears that the dynamics of feral alfalfa is seed limited in mown strips and recruitment limited in non-mown strips.

The elasticity analysis has revealed that management strategies that act to solely suppress adult survival may lead to complex, potentially chaotic and unpredictable dynamics with high population densities, contrary to the management objective.

In this regard, Buckley et al. [43] stated that the effects of adult mortality achieved by management approaches may be offset by greater establishment of seedlings or by greater fecundity of the escapes. Similar observations were also noted by other researchers [34], [44]. Thus, management efforts exclusively focused on controlling adult plants may not be effective for managing roadside alfalfa populations.

Analysis of the model’s behavior suggests that a simple and practicable option to managing roadside alfalfa populations is to implement a timely-mowing regime, which can prevent seed production entirely. For southern Manitoba, a single roadside mowing annually during early- to mid- August will be sufficient to prevent seed production in most feral alfalfa plants. Currently, complete mowing of road-verges does not occur in southern Manitoba because it is neither mandated nor is it practically feasible. In areas where roadside are mown, the timing of mowing (second pass) can vary from late August to late September, allowing for seed production in feral alfalfa populations. In regions where roadverge mowing is already practiced, feral alfalfa populations could be effectively managed by merely altering the time of mowing. Targeted herbicide applications, which can provide a total control of seedling, rosette and adult stages will also be effective; however, the presence of dormant seedbank may contribute to new recruits over time. If total control of a population is warranted, repeated herbicide applications are required at the shortest possible regeneration time (2 years) until the exhaustion of the seedbank population (about 7 years, data not shown). Timely mowing until the exhaustion of the seedbank, followed by herbicide application to control the rest of the demographic stages may also be a useful strategy. Programs can be designed in collaboration with municipality weed supervisors and roadverge managers to achieve the management goals.

Although random seed spill or seed escape through farming operations may not substantially influence existing feral populations, any such seed input may found new feral populations and maintain existing populations. Therefore, adequate attention should be paid to prevent anthropogenic seed escape into roadside habitats, especially in areas where gene flow is a concern. Furthermore, intentional planting of alfalfa in roadside habitats should be discouraged in such regions. Given the existence of strong density-dependent effects, auto-allelopathy and low seed dispersal, it is less likely that feral alfalfa populations will be invasive in roadside habitats.

We developed a static model, which was parameterised based on the growth conditions observed during the study years. Consequently, the model does not consider the effects of environmental stochasticity on feral population dynamics. Thus, the long-term dynamics, particularly the extinction probabilities and the time taken to reach equilibrium could have been different under varying environmental conditions. Nevertheless, this model confirms that alfalfa is capable of establishing self-sustaining populations in roadside habitats, which has implications for preventing the persistence of GE traits in the environment. In the context of novel trait confinement, the degree to which feral populations need to be managed will be dictated by the tolerance level established for specific traits in specific production systems. Landscape-level gene flow models will be useful for making management decisions to meet the threshold requirements for various production systems, and our findings will be valuable in developing such models for alfalfa. Additionally, the modelling approach and the concepts used in this study could be extended to other perennial plants with comparable life-history characteristics.
